# Combination of Side-Swing Flap With Negative-Pressure Wound Therapy Is Superior to Open Excision or Flap Alone in Children With Pilonidal Sinus—But at What Cost?

**DOI:** 10.3389/fped.2021.595684

**Published:** 2021-04-14

**Authors:** Deborah Dorth, Ingo Königs, Julia Elrod, Tarik Ghadban, Konrad Reinshagen, Michael Boettcher

**Affiliations:** ^1^Department of Pediatric Surgery, University Medical Center Hamburg-Eppendorf, Hamburg, Germany; ^2^Department of General, Visceral and Thoracic Surgery, University Medical Center Hamburg-Eppendorf, Hamburg, Germany

**Keywords:** pilonidal sinus, negative-pressure wound therapy, NPWT, open excision, side-swing flap

## Abstract

**Background:** Pilonidal sinus (PS) disease frequently occurs in adolescents and young adults, and in many cases involves wide excision or local flaps as treatment. These treatments are associated with a significant recurrence rate, a long healing time, and thus absence from school or work. The hybrid technique, which is a combination of side-swing plasty with negative-pressure wound therapy (NPWT) may improve these outcomes. The aim of the study was to compare the latter with other current methods.

**Methods:** Children presenting with a pilonidal sinus to two referral centers for pediatric surgery from January 2017 till June 2019 and subsequent (1) slide-swing plasty, (2) open excision, or (3) slide-swing plasty in combination with NPWT were included in this retrospective study. Type of therapy, number of interventions, duration of hospitalization, complications, and recurrence rate were recorded. In addition, data was retrieved from the national diagnosis-related group for inpatient statistics, for all patients who underwent surgery for pilonidal sinus in 2015 and 2016.

**Results:** In total, 85 children were included, with a mean age of 15 years and a near equal gender distribution (53% female). The minimum follow-up was 1 year. In 56% open resection was performed, while 18% underwent a slide-swing plasty and 26% a slide-swing plasty in combination with NPWT. While the hybrid technique was superior regarding recurrence rate in comparison to open excision (24 vs. 5%, *p* = 0.047), it had significantly longer hospital stay [17.41 (15.63) vs. 3.65 (1.68) days, *p* < 0.001] and number of interventions [4.14 (4.07) vs. 1.04 (0.29), *p* < 0.001].

**Conclusions:** Management of PS disease using slide-swing plasty in combination with NPWT is an effective treatment and is associated with low recurrence rate and minimal morbidity. However, this type of treatment is accompanied by an elongated hospitalization time and more frequent interventions. A diligent case by case evaluation and thorough patient counseling is thus necessary when choosing the right technique for the treatment of PS disease.

## Background

Pilonidal sinus (PS) disease was first described by Mayo in 1833 as a hair-containing cyst located just above the coccyx (1). PS disease remains a common condition affecting mainly the sacrococcygeal region in the midline natal cleft, and occurs more frequently in young male subjects, with a male to female ratio of 3:1 (2). Symptomatic PS cysts may cause significant discomfort, pain, and immobility, often requiring multiple surgical interventions under general anesthesia as treatment, which are associated with prolonged absences from school or work (3).

Pathogenesis of PS cysts is still not fully understood. As such, one main theory is known as the theory of follicular occlusion tetrad, which believes that the underlying pathophysiology of PS disease involves an occlusion of the infundibular hair follicle on account of follicular hyperkeratosis as the first step. As a consequence, the clogged follicle ignites and subsequently bursts, leading to inflammation of the surrounding subcutaneous tissue (4). However, the most accepted theory by Karydakis suggests that scalp or local hair penetrates into the subcutaneous tissue causing a localized foreign body reaction resulting in the formation of a cyst and subsequent abscess formation (5). This theory is reinforced by the fact that especially hairdressers have been shown to develop formations similar to PS cysts interdigitally on their hands and feet with large quantities of cut hair fragments found in these pilonidal nests (6). Moreover, another study assessing the length and morphology of hair found within PS cysts revealed that the hairs were shorter than other bodily hair. Lending support to this theory is the fact that hair found within PS cysts resembled cut hair rather than body hair as visualized using electron microscopy, thus further supporting the Karydakis theory (7).

Management of PS disease should ideally be simple, attaining low recurrence rates with the least possible tissue trauma. While surgery is considered to be the most successful treatment for chronic PS disease, it still leads to a recurrence rate of 10% (8). The most common procedure is the open excision which comprises resection of the pori including the fox-like fistula tract with its surrounding soft tissue. With respect to recurrence rates of the PS cyst using this procedure, a recent meta-analysis showed a 1% chance of return after 1 year, while this percentage increased to 3.2% after 2 years and 16.5% after 5 years (9). One perk of open excision for PS treatment is the fact that it is a technically simple intervention that can be carried out as an outpatient procedure. However, this method has the longest wound healing time of ~3 months, resulting in patients missing school or work for an average of 1 month. Another positive aspect of open excision is the rare development of seroma formation. Baring this in mind, open excision treatment places high demands on the patient, who must be able to independently wash and dress the wound at home (9–12). The second most common procedure for treatment of PS cysts is primary wound closure including lateralization of the seam after the excision using various techniques i.e., Karydakis, Limberg, Schrudde-Olivari flap. Using this technique, a meta-analysis revealed a recurrence rate of 1% after 1 year, 1.6% after 2 years, and 6.7% after 10 years (9). Moreover, post-operative wound healing time is faster in comparison to open excision, taking around 2–3 weeks, and seromas occur seldomly (9, 13, 14). As a third option for treatment of PS disease a more recent approach involves negative-pressure wound therapy (NPWT). Especially in complex cases involving recurring PS disease (15–18). Until today, the combination of side-swing plasty with negative-pressure wound therapy and secondary closure has not been evaluated for PS disease patients, making it the aim of the current study.

## Materials and Methods

Retrospective analysis of all children and adolescents treated with pilonidal sinus abscess formation at two large departments for pediatric surgery between January 2017 and June 2019, who met the following inclusion criteria: side-swing plasty (Schrudde-Olivari flap) with or without primary NPWT or open excision without NPWT. Demographic data was obtained on age and gender, as well as disease-related data, namely (1) immunodepression, (2) diabetes, (3) previous excision of the pilonidal sinus cyst, (4) complications, (5) recurrence, (6) duration of hospital stay, and (7) number of interventions.

### National Diagnosis-Related Group Inpatient Statistics

Moreover, a controlled remote analysis of the nationwide Diagnosis Related Group (DRG) database from 2016 to 2017 was undertaken to evaluate inpatient data provided by the Research Data Centers of the German Federal Statistical Office and the Statistical Offices of the Länder. The German adaptation of the International Classification of Diseases (ICD) Tenth Revision and the procedure coding system (Operationen-und Prozedurenschlüssel, OPS) were used to identify diagnoses and procedures. Every inpatient case with a procedure code for surgical treatment of pilonidal sinus abscess formation was included. The diagnosis codes L050 and L059 and the procedure code 5-897 were applied.

### Statistics

All data were analyzed with SPSS Statistics 26 (IBM, NY, USA) and GraphPad Prism 8 (GraphPad, CA, USA). Differences between groups were calculated using the *t*-test and ANOVA, whenever applicable. Data are presented as mean ± standard deviation (SD). The level of significance was set at 0.05.

## Results

In total 85 children with PS disease presented at the department of pediatric surgery of the University Medical Center Hamburg-Eppendorf and the Children's Hospital Altonaer Kinderkrankenhaus between January 2017 and June 2019. One patient was excluded in which open excision with primary NPWT was performed. The minimum follow-up time was 1 year, while the mean age of the enrolled patients was 14.72 (1.90) years. Unlike other studies, which show that PS disease is more prevalent in males, 52.9% of the study participants in our study were female. With regards to demographics, no significant differences between all groups were found for average age and gender distribution. Moreover, the number of previous resections, immunosuppression, and diabetes did not differ among the three groups (Table 1).

**Table 1:** Patients characteristics.

	**Open**	**Flaps**	**Hybride**	***p***
Number	48/85	15/85	22/85	–
Age in years (SD)	14.73 (1.30)	14.67 (3.22)	14.73 (1.92)	n.s.
Gender (female)	25/48	6/15	14/22	n.s.
Previous resection	1/48	2/15	4/22	n.s.
Immunosuppression	1/48	0/15	2/22	n.s.
Diabetes	2/48	2/15	0/22	n.s.

Overall, 48 (56%) open excisions were performed and compared to 15 (18%) side-swing plasties (Schrudde-Olivari flap) and 22 (26%) side-swing plasties with primary NPWT (hybrid technique).

Regarding perioperative outcome significant differences between groups were found: (1) Seroma formation was rarer in the open excision group, as well as the group that underwent side-swing plasty without NPWT; As such, patients in the hydride group suffered significantly more from seroma formation in comparison to patients with open excision (*p* < 0.001), whereas only non-significant differences were found when compared to the flap only group (Table 2). (2) While the rate of wound infections was similar in all groups (Table 2), differences between groups regarding recurrence rate were seen. More specifically, a relapse was found in 23% of the patients undergoing open excision and in 26% of the patients with side-swing plasty. In the hydride group the rate of recurrence was 9%, which is significantly lower compared to the non-hybrid groups (*p* = 0.007). (3) The number of interventions necessary in order for complete wound closure to occur differed between groups. In the hybrid group four interventions were needed compared to one in the open excision (*p* = 0.001) and two and a half in the side-swing plasty group (*p* > 0.05). (4) Lastly, while there were no cases of death, bleeding, peritonitis, or rectal fistula in the current study, the hospitalization length in days was significantly shorter for patients assigned to the non-hybrid groups compared to the hybrid group (*p* = 0.004).

**Table 2:** Outcome parameters.

	**Open**	**Hybrid**	***p***
Seroma	0/48	6/22	<0.001
Infection	4/48	1/22	n.s.
Recurrence	11/48	1/22	0.047
Interventions (SD)	1.04 (0.29)	4.14 (4.07)	<0.001
Hospitalization (SD)	3.65 (1.68)	17.41 (15.63)	<0.001
	**Open**	**Flap**	***p***
Seroma	0/48	2/15	n.s.
Infection	4/48	1/15	n.s.
Recurrence	11/48	4/15	n.s.
Interventions (SD)	1.04 (0.29)	2.47 (2.85)	0.001
Hospitalization (SD)	3.65 (1.68)	8.73 (10.49)	0.002
	**Flap**	**Hybrid**	***p***
Seroma	2/15	6/22	n.s.
Infection	1/15	1/22	n.s.
Recurrence	4/15	1/22	n.s.
Interventions (SD)	2.47 (2.85)	4.14 (4.07)	n.s.
Hospitalization (SD)	8.73 (10.49)	17.41 (15.63)	n.s.
	**Non-hybrid**	**Hybrid**	***p***
Seroma	2/63	6/22	0.003
Infection	5/63	1/22	n.s.
Recurrence	15/63	1/22	n.s.
Interventions (SD)	1.38 (1.51)	4.14 (4.07)	0.007
Hospitalization (SD)	4.86 (5.63)	17.41 (15.63)	0.004

### National Diagnosis-Related Group Inpatient Statistics

The nationwide database analysis showed that in 2 years 10,528 procedures for PS disease were performed in patients aged 0–18 in Germany. Of these, 59.3% were male. Mean age was 16.08 (2.48), which is more in line with the known gender distribution for the disease. The mean hospitalization time was 2.48 (3.07) days and no patient died during the duration examined for this study. Moreover, no patients developed peritonitis, while 24 cases (0.2%) did develop a rectal fistula. No bleeding complications were documented.

## Discussion

The nationwide database underlines the relevance of PS disease in children, recording more than 5,000 procedures annually. As such, it is one of the most frequently performed procedures in children. Nevertheless, optimal treatment of PS disease remains controversial and the existing techniques are far from perfect. Therapy for PS cysts should not only be effective but also simple. Procedures should aim to reduce hospitalization time to a minimum, furthermore attaining quick recovery for patients and resumption of normal activities (19). While the hybrid technique assessed in this study offers very low rate of infections and recurrence, the costs of this technique are very high: the number of interventions was three times greater and the hospitalization rate almost four times as high as in the non-hydride group. Additionally, NPWT itself is very expensive due to the material necessary to conduct the therapy, and thus may not be applicable in other clinical settings.

Data obtained from the nationwide analysis indicates that the average length of hospitalization lies at 2.48 days – a very short period of time compared to data acquired in our study. It is therefore to be presumed, that most patients were treated with the open excision technique, which was evaluated to have the shortest hospitalization rate in days in our study. However, in the absence of exact information from the database, a detailed comparison with our data cannot be conducted.

Due to the very promising results of the hybrid technique with respect to relapse and infection rate on the one hand, but the substantial costs and long hospitalization time on the other hand, we believe that a diligent case by case consideration is necessary when choosing the right procedure for each individual presenting with a symptomatic PS cyst. Thus, when deciding which procedure to perform, evaluation should not only focus on objective criteria, such as the extend of the abscess, the exact anatomical location, or the medical history of the patient (i.e., passed PS abscesses), but also subjective criteria, such as the patient's preferences, compliance, and domestic support for aftercare. Thorough patient counseling therefore is indispensable. Furthermore, we believe that in certain cases when high patient compliance and home care support are available, maintaining NPWT as an outpatient procedure could be advantageous for the patient, as hospitalization time could be reduced to a minimum. However, at the moment implementation of this option is difficult in Germany due to bureaucratic reasons.

A limitation of this study is the relatively short-term follow-up period of only 1 year (longer in some cases), which might not provide enough time in order to properly assess recurrence and complication rates. Thus, additional studies with longer follow-up periods are necessary as to ascertain the efficacy of this hybrid technique.

Management of symptomatic PS cysts using slide-swing plasty in combination with NPWT is an effective and simple treatment that is associated with low recurrence rate and minimal morbidity. Despite being expensive and resulting in numerous interventions. Additionally, the currently employed wide open excisional or local flap treatments for PS disease pose a heavy burden on the patient, since these interventions are often accompanied by a lengthy recovery time. As the patient population is quite young, lengthy hospitalization is thus often associated with an absence from normal everyday activities resulting in a decreased quality of life (20). In order to minimize the physical and economic impact of PS disease treatment and further maintain an adequate quality-of-life, many adolescents and young adults are looking for less invasive techniques to treat their PS disease. Hence, after this analysis, we switched to the GIPS procedure, a minimally invasive technique, with very short anesthesia time. It can be conducted as an out-patient procedure and is associated with minimal trauma, and low complication and recurrence rates (21, 22). In addition, studies have found that the procedure results in a lower pain experience and patients can return to work and everyday life more quickly. Another minimally invasive surgery option would be the conduction of an endoscopic treatment, which is reported to have very low recurrence and fast recovery rates (23). However, these methods may not be suitable for abscessed pilonidal sinus, recurrent PS disease and extensive findings. If the previously described findings are present, we recommend abscess drainage and secondary surgery.

## Conclusion

In conclusion management of symptomatic PS cysts using slide-swing plasty in combination with NPWT is an effective treatment that is associated with low recurrence rate and minimal morbidity. However, this type of treatment is accompanied by a very long hospitalization time and frequent interventions. As such, we recommend a diligent case by case evaluation and thorough patient counseling when choosing the right surgical technique for treatment of PS disease. Hence, future studies should compare the hybrid technique with other promising interventions like the GIPS procedure or endoscopic treatment.

## Data Availability Statement

The original contributions presented in the study are included in the article/supplementary material, further inquiries can be directed to the corresponding author/s.

## Ethics Statement

Ethical review and approval was not required for the study on human participants in accordance with the local legislation and institutional requirements. Written informed consent from the participants' legal guardian/next of kin was not required to participate in this study in accordance with the national legislation and the institutional requirements.

## Author Contributions

DD, IK, and JE designed the study, collected data, analyzed the data, and drafted and revised the paper. TG collected data, analyzed the data, and revised the paper. KR collected data, and revised the paper. MB designed the study, collected data, analyzed the data, performed statistics and drafted and revised the paper. All authors contributed to the article and approved the submitted version.

## Conflict of Interest

The authors declare that the research was conducted in the absence of any commercial or financial relationships that could be construed as a potential conflict of interest.
